# Prevalência em Evidência: As Valvopatias que Dominam o Cenário Brasileiro

**DOI:** 10.36660/abc.20260464

**Published:** 2026-07-14

**Authors:** Ana Teresa Timóteo

**Affiliations:** 1 Hospital Santa Marta Serviço de Cardiologia Lisboa Portugal Serviço de Cardiologia, Hospital Santa Marta, ULS São José, Lisboa – Portugal; 2 NOVA Medical School Lisboa Portugal NOVA Medical School, Lisboa – Portugal

**Keywords:** Valvopatia, Prevalência, Brasil

A doença valvar é uma patologia cardiovascular muito prevalente, estimando-se que atinja mais de 2% da população, e está associada ao aumento da mortalidade.^1^ No entanto, a maioria dos estudos epidemiológicos é realizada em países de alta renda, e sabemos que as características variam de acordo com o país e a região do mundo. De fato, em países de alta renda, predomina a doença valvar degenerativa, enquanto nos países de renda mais baixa a patologia reumática e infecciosa ainda é muito prevalente. As condições socioeconômicas também têm implicações importantes no diagnóstico e no tratamento dessa patologia.

Dados do *EURObservational Research Programme Valvular Heart Disease* II (EORP-VHD II) *Survey*, com participantes de 28 países europeus, com idade média de 71 anos, relataram a estenose valvar aórtica como a doença valvar mais frequente, presente em 41% dos pacientes, seguida à distância pela regurgitação mitral, regurgitação aórtica e estenose mitral.^2^ A etiologia degenerativa foi a mais comum, com exceção da estenose mitral, em que a etiologia reumática predominou. Esse registro reforçou os resultados do EORP-VHD inicial, realizado em 2001.^3^ O aumento da estenose aórtica, será provavelmente consequência do envelhecimento populacional, e a redução da estenose mitral reumática, resultante da diminuição da febre reumática na Europa. Dados publicados recentemente no ATLAS da *European Society of Cardiology* também destacam que as doenças valvares, continuam aumentando em prevalência e são responsáveis por milhares de mortes anualmente.^4^

O artigo desta edição dos Arquivos Brasileiros de Cardiologia, de Schmitz et al., busca avaliar o peso da doença valvar e sua associação com mortalidade no Brasil.^5^ Para isso, analisa a coorte do Estudo Longitudinal de Saúde de Adultos – Brasil (ELSA-Brasil), que investigou a presença de doenças cardiovasculares e diabetes em funcionários de instituições de ensino ou pesquisa. Para este estudo, foram selecionados os indivíduos que realizaram ecocardiograma transtorácico. Trata-se de uma coorte de grandes dimensões (3.267 participantes) e representativa do Brasil, uma vez que foi conduzida em seis estados. Os pacientes foram divididos de acordo com a gravidade da doença valvar (nenhuma, leve e moderada/grave). O grupo moderada/grave era composto por indivíduos mais velhos, com mais comorbidades, e a doença valvar mais frequente foi a regurgitação mitral, seguida pela regurgitação aórtica e tricúspide. É também um grupo caracterizado por maior remodelamento cardíaco. Esse grupo apresentou risco elevado de mortalidade em 10 anos (HR 3,7), independentemente da idade, sexo e hipertensão. A regurgitação tricúspide foi mais associada ao sexo feminino.

Este estudo é muito relevante, e os autores demonstraram que, no Brasil, a doença valvar é muito frequente na comunidade, aumenta com a idade e que a presença de doença moderada/grave está associada a mais comorbidades, maior remodelamento cardíaco, pior função sistólica e impacto na mortalidade a longo prazo. Ressaltamos, contudo, que esta análise apresenta algumas limitações que podem influenciar a interpretação.

Por um lado, o grupo com doença moderada/grave representa apenas 2,4% dos participantes, ou seja, apenas 70 indivíduos. Assim, o poder estatístico é bastante limitado e não permitiu melhor caracterização de cada uma das doenças valvares encontradas. Outra limitação importante decorre da população selecionada para o estudo. São, sobretudo, funcionários de instituições e apenas até 75 anos. Por esse motivo, indivíduos de meios sociais mais desfavorecidos ou mais idosos não foram incluídos. De fato, a idade média dos participantes foi de 61 anos. Trata-se, portanto, de uma população selecionada, e os resultados poderiam ser diferentes se a amostra fosse mais ampla e representativa. Isso é particularmente evidente nos resultados apresentados, em que a estenose valvar aórtica não apresenta prevalência relevante, ao contrário de muitas outras séries. Isso pode refletir uma característica específica do país, mas é facilmente explicado pela ausência de pacientes muito idosos (> 75 anos) na coorte analisada.

Outra dúvida que pode ser levantada diz respeito ao seguimento dos pacientes com doença valvar moderada/grave. É plausível que alguns desses pacientes tenham sido submetidos a intervenção ao longo do seguimento (cerca de 10 anos), e não está claro se esses indivíduos foram excluídos da análise a partir do momento da intervenção. Esse é um fator de viés importante na interpretação dos resultados.

Em conclusão, este importante subestudo do registro ELSA-Brasil permite caracterizar melhor a prevalência da doença valvar no Brasil e demonstra o impacto prognóstico da doença valvar moderada/grave. Contudo, as limitações mencionadas recomendam cautela na generalização dos resultados apresentados.
