# Supplementary material for: Prevalência em Evidência: As Valvopatias que Dominam o Cenário Brasileiro
Source: Arq Bras Cardiol. 2026 Jul 14;123(6):e20260464. [Article in Portuguese] doi: 10.36660/abc.20260464 (PMC13399143; doi:10.36660/abc.20260464)
Supplement: Translation

# Translation: Prevalence in Evidence: Valvular Heart Diseases Dominating the Brazilian Scenario

**DOI:** 10.36660/abc.20260464i

**Authors:** Ana Teresa Timóteo

**Affiliations:** 1 Lisboa Portugal Serviço de Cardiologia, Hospital Santa Marta, ULS São José, Lisboa – Portugal; 2 Lisboa Portugal NOVA Medical School, Lisboa – Portugal

**Keywords:** Valvular Heart Disease, Prevalence, Brazil

Valvular heart disease is a highly prevalent cardiovascular condition, estimated to affect more than 2% of the population, and is associated with increased mortality.^1^ However, most epidemiological studies are conducted in high-income countries, and we know that the characteristics vary according to the country and region of the world. In fact, degenerative valvular heart disease predominates in high-income countries, while rheumatic and infectious diseases are still very prevalent in lower-income countries. Socioeconomic conditions also have important implications for the diagnosis and treatment of this condition.

Data from the EURObservational Research Programme Valvular Heart Disease II (EORP-VHD II) Survey, with participants from 28 European countries and an average age of 71 years, reported aortic valve stenosis as the most frequent valvular disease, present in 41% of patients, followed at a distance by mitral regurgitation, aortic regurgitation, and mitral stenosis.^2^ Degenerative etiology was the most common, except for mitral stenosis, where rheumatic etiology predominated. This record reinforced the results of the initial EORP-VHD, conducted in 2001.^3^ The increase in aortic stenosis is likely a consequence of population aging, and the reduction in rheumatic mitral stenosis is a result of the decrease in rheumatic fever in Europe. Data recently published in the ATLAS of the European Society of Cardiology also highlight that valvular diseases continue to increase in prevalence and are responsible for thousands of deaths annually.^4^

The article in this edition of the *Arquivos Brasileiros de Cardiologia*, by Schmitz et al., aims to evaluate the burden of valvular heart disease and its association with mortality in Brazil.^5^ To this end, it analyzes the cohort of the Longitudinal Study of Adult Health – Brazil (ELSA-Brasil), which investigated the presence of cardiovascular diseases and diabetes in employees of teaching or research institutions. For this study, individuals who underwent transthoracic echocardiography were selected. This is a large cohort (3,267 participants) and representative of Brazil, as it was conducted in six states. Patients were divided according to the severity of valvular heart disease (none, mild, and moderate/severe). The moderate/severe group consisted of older individuals with more comorbidities, and the most frequent valvular heart disease was mitral regurgitation, followed by aortic and tricuspid regurgitation. This group is also characterized by greater cardiac remodeling. This group presented a high risk of mortality in 10 years (HR 3.7), regardless of age, sex, and hypertension. Tricuspid regurgitation was more associated with females.

This study is very relevant, and the authors demonstrated that, in Brazil, valvular heart disease is very frequent in the community, increases with age, and that the presence of moderate/severe disease is associated with more comorbidities, greater cardiac remodeling, worse systolic function, and an impact on long-term mortality. However, we emphasize that this analysis has some limitations that may influence its interpretation.

On the one hand, the group with moderate/severe disease represents only 2.4% of the participants, that is, only 70 individuals. Thus, the statistical power is quite limited and does not allow for a better characterization of each of the valvular diseases found. Another important limitation stems from the population selected for the study. They are mainly employees of institutions and are only up to 75 years old. For this reason, individuals from more disadvantaged social backgrounds or older individuals were not included. In fact, the average age of the participants was 61 years. It is, therefore, a selected population, and the results could be different if the sample were larger and more representative. This is particularly evident in the results presented, where aortic valve stenosis does not show a relevant prevalence, unlike many other series. This may reflect a specific characteristic of the country, but it is easily explained by the absence of very elderly patients (> 75 years) in the analyzed cohort.

Another question that can be raised concerns the follow-up of patients with moderate/severe valvular disease. It is plausible that some of these patients underwent intervention during the follow-up period (approximately 10 years), and it is unclear whether these individuals were excluded from the analysis at the time of intervention. This is an important bias factor in the interpretation of the results.

In conclusion, this important sub-study of the ELSA-Brasil registry allows for a better characterization of the prevalence of valvular heart disease in Brazil and demonstrates the prognostic impact of moderate/severe valvular heart disease. However, the limitations mentioned recommend caution in generalizing the results presented.
